# Letter to the Editor regarding “Prognostic value of age and Wassel classification in the reconstruction of thumb duplication”

**DOI:** 10.1007/s11832-014-0591-2

**Published:** 2014-05-13

**Authors:** Robert R. Dijkman, Christianne A. van Nieuwenhoven, Steven E. R. Hovius

**Affiliations:** 1 Department of Plastic and Reconstructive Surgery and Hand Surgery, Erasmus MC, University Medical Center Rotterdam, Room Ee 15.91, PO Box 2040, 3000 CA Rotterdam, The Netherlands; 2 Department of Plastic and Reconstructive Surgery and Hand Surgery, Erasmus MC, University Medical Center Rotterdam, Room HS 511, PO Box 2040, 3000 CA Rotterdam, The Netherlands; 3 Department of Plastic and Reconstructive Surgery and Hand Surgery, Erasmus MC, University Medical Center Rotterdam, Room HS 501, PO Box 2040, 3000 CA Rotterdam, The Netherlands

Sirs,

With great interest we have read the article “Prognostic value of age and Wassel classification in the reconstruction of thumb duplication” by Dr. M. Cabrera González and colleagues [1]. We would like to congratulate the authors with this publication and thank them for posing a relevant question. However, we feel there are a number of debatable issues related to the manner in which the prognostic factors were evaluated.

Our first concern regards the mean duration of follow-up in relation to the mean age at surgery and following complications. The authors state that there were no differences in follow-up among the Wassel groups or in relation to other factors. However, there is no reference to mean age at follow-up. Moreover, the mean follow-up of 44.1 months, with the range 3–144 months, and the mean age of 20 months at surgery, with 70 % of the study population undergoing surgery before the age of 19 months, suggest skewed distributions towards early surgery with short follow-up.

This is problematic since complications may become apparent at a later age. Clinodactyly (the main complication in this study) in particular may develop during growth spurts due to damage or asymmetry of joints and bone or because of tendon imbalance. This condition might also appear over time due to decreasing volume of the tissue envelope of the thumb. Therefore, the suggested relationship between early surgery and reduced complication rate may very well be confounded by the short follow-up in the early surgery group.

Another concern we have is the use of the Tada system to define outcome. Contrary to the authors’ suggestion, at least ten other systems are available to describe outcome in radial polydactyly. Overall outcome depends on the system of choice. For example, Ogino’s modification of the Tada system is much more stringent on the range of motion (ROM) (2 points for ROM ≥131° vs. 2 points for ROM ≥70°). In addition, no ‘poor’ results were found in this study according to the Tada system, while the results show a 27 % complication and 12 % reoperation rate. This implies that the Tada system was not sufficiently sensitive to detect complications requiring revision surgery as poor outcome.

Furthermore, it is questionable whether the Tada system encompasses all relevant items. The authors rightfully point out that the Tada system is solely based on function, and they also state that it encompasses the most limiting complications (i.e. clinodactyly and stability). However, it is unknown how thumb misalignment and instability affect children’s hand function. Moreover, it is plausible that patients care more about postoperative appearance than about function, which may even influence their perception on how their hand functions. Consequently, a greater emphasis on aesthetic parameters as part of the outcome assessment is warranted.

In summary, the authors’ choice of the Tada system as primary outcome affects the results the the study and the conclusions which can be drawn from these results.

As a final note, the classification system depicted in Fig. 3 [1] is not the original Wassel classification, as type VII does not appear to be of the triphalangeal kind. Instead, it resembles a ‘floating’ connection at the metacarpophalangeal level, a type excluded from the analysis according to the Methods section [1]. However, results for this ‘type VII’ were presented with a 0 % complication rate. On the other hand, triphalangism was excluded from the analysis in accordance with the Methods section. Complications linked to polydactyly with triphalangial thumbs is one of the reasons they are often described and treated as a distinct entity within the spectrum. In conclusion, analysis of triphalangism instead of floating components would have been adherent to the Wassel classification, although it might have led to a higher complication rate.

Although the authors discuss their results in a nuanced way, we hope they can further address the above-mentioned concerns. We feel the retrospective nature of the study combined with the other methodological concerns undermine the conclusions drawn by the authors. In our opinion, the evidence in this article does not prove patients with radial polydactyly should undergo surgery at a very young age to reduce complications, regardless of Wassel type.

Sincerely,

The authors
